# Effects of Propidium Monoazide (PMA) Treatment on Mycobiome and Bacteriome Analysis of Cystic Fibrosis Airways during Exacerbation

**DOI:** 10.1371/journal.pone.0168860

**Published:** 2016-12-28

**Authors:** Linh Do Ngoc Nguyen, Pieter Deschaght, Sophie Merlin, Alexandre Loywick, Christophe Audebert, Sabine Van Daele, Eric Viscogliosi, Mario Vaneechoutte, Laurence Delhaes

**Affiliations:** 1 Institut Pasteur de Lille, Center for Infection and Immunity of Lille (CIIL), INSERM U1019, CNRS UMR 8204, University of Lille, Lille, France; 2 Laboratory for Bacteriology Research, Faculty of Medicine & Health Sciences, Ghent University, Ghent, Belgium; 3 GenesDiffusion, Douai, France; 4 PEGASE, Biosciences, Institut Pasteur de Lille, Lille, France; 5 Department of Pediatrics and Genetics, Faculty of Medicine & Health Sciences, Ghent University, Ghent, Belgium; 6 Parasitology-Medical Mycology Department, Regional Hospital Center, Faculty of Medicine, Lille, France; Lee Kong Chian School of Medicine, SINGAPORE

## Abstract

**Introduction and Purpose:**

Propidium monoazide (PMA)-pretreatment has increasingly been applied to remove the bias from dead or damaged cell artefacts, which could impact the microbiota analysis by high-throughput sequencing. Our study aimed to determine whether a PMA-pretreatment coupled with high-throughput sequencing analysis provides a different picture of the airway mycobiome and bacteriome.

**Results and Discussion:**

We compared deep-sequencing data of mycobiota and microbiota of 15 sputum samples from 5 cystic fibrosis (CF) patients with and without prior PMA-treatment of the DNA-extracts. PMA-pretreatment had no significant effect on the entire and abundant bacterial community (genera expressed as operational taxonomic units (OTUs) with a relative abundance greater than or equal to 1%), but caused a significant difference in the intermediate community (less than 1%) when analyzing the alpha biodiversity Simpson index (p = 0.03). Regarding PMA impact on the airway mycobiota evaluated for the first time here; no significant differences in alpha diversity indexes between PMA-treated and untreated samples were observed. Regarding beta diversity analysis, the intermediate communities also differed more dramatically than the total and abundant ones when studying both mycobiome and bacteriome. Our results showed that only the intermediate (or low abundance) population diversity is impacted by PMA-treatment, and therefore that abundant taxa are mostly viable during acute exacerbation in CF. Given such a cumbersome protocol (PMA-pretreatment coupled with high-throughput sequencing), we discuss its potential interest within the follow-up of CF patients. Further studies using PMA-pretreatment are warranted to improve our “omic” knowledge of the CF airways.

## Introduction

Cystic fibrosis (CF) is associated with severe lung damage because of reduced mucociliary clearance and subsequent polymicrobial infections [1]. Approximately 90% of CF patients suffer from lung destruction, promoted by pathogens such as *Pseudomonas aeruginosa*. Consequently, antibiotic treatment represents a keystone of CF therapy, preventing chronic infection and reducing exacerbation rates and alteration of pulmonary function.

Until now, strategies to manage CF lung disease principally consist of routine microbiology (including microbiological culture of sputum samples) and appropriate antibiotic treatment [1,2]. While these conventional methods identify viable and abundant pathogens, they could not detect uncultivable or difficult-to-cultivate microorganisms. The recent use of culture-independent methods based on high-throughput sequencing (HTS) has provided a more complete view of the CF lung bacterial microbiome and its evolution during respiratory alteration [3–7]. However, the current use of HTS does not differentiate the DNA of living microorganisms and that of dead microorganisms in sputum samples, which might be essential in the clinical context of exacerbation. Sample pretreatment with propidium monoazide (PMA) might facilitate viable microorganism detection, as recently proposed [6].

PMA is a chemical compound that selectively penetrates into cells with damaged membrane (dead cells), intercalates covalently into their DNA and inhibits PCR amplification of these dead microorganisms. It has been combined with different molecular methods to remove bias from dead or damaged cell artefacts in microbial samples [6,8–17]. Exclusively several studies have shown that the PMA cross-linking method leads to changes in the bacterial communities using HTS [6,11–15].

The aim of the present study was to evaluate whether PMA-pretreatment associated with HTS analysis is able to provide a more realistic profile of the viable fungal and bacterial communities in the CF airways, and consequently reflect more closely the clinical outcome of the patients. We compared HTS data of pro- and eukaryotic microbiota obtained with and without PMA-pretreatment of DNA extracts from sputum samples, in order to assess the impact of PMA-pretreatment when quantifying the respiratory mycobiome and bacteriome of CF patients.

## Materials and Methods

### Ethics agreement

Ethics approval for this study was provided by the Ethics Committee of Ghent University Hospital, Belgium (project nr. 2007/503). Study was performed in accordance with approved national and international guidelines; written informed consent was obtained from all the patients > 18 years or from the parents and children older than 12 years, as previously reported [16,17].

### Patients and sampling

Fifteen sputum samples were prospectively collected from 5 CF patients (3 homozygous and 2 heterozygous carriers of F508del-CFTR) followed at Ghent Hospital for acute pulmonary exacerbation. Patients aged from 15 to 34 years old, were chronically colonized with *P*. *aeruginosa* [1], and received the same antimicrobial treatment: Tazocin^®^ (Piperacillin/Tazobactam 4000mg/500mg) by intravenous administration during 15 days. Sputum samples were collected as previously reported [16,17].

### PMA pretreatment

The PMA-pretreatment and DNA extractions were conducted at the laboratory for Bacteriology Research of Ghent University as previously reported [16,17]. Briefly, each sputum sample was transferred into 2 wells of a 24-well plate and 10 μl of PMA (final concentration: 50 mM) (Biotium, Hayward, CA) was added to a first 190 μl sample aliquot (for PMA-qPCR) and 10 μl saline buffer was added to the other sample aliquot. After 30 min incubation in the dark on a shaker, the samples were exposed to a 500 W halogen light source for 10 min at a distance of 20 cm. During exposure, the 24-well plate was kept on ice to avoid overheating of the samples.

### Culture-based quantification of P. aeruginosa [16,17]

Homogenized sputum samples were diluted serially tenfold in physiological saline. Each dilution (25μl) was inoculated in triplicate on cetrimide agar plates. The *P*. *aeruginosa* load was determined after 72 h of incubation at 37°C in ambient atmosphere.

### Quantitative PCR targeting either *P*. *aeruginosa or A*. *fumigatus*

After DNA extraction using the easyMAG Nuclisens DNA extractor (bioMérieux, Marcy-l’Etoile, France), qPCR was carried out on the Light-Cycler480 (Roche, Basel, Switzerland) as previously described [16,17]. The reaction mixture contained 5 μl of Probes Master kit, 0.5 μM of each primer (*P*. *aeruginosa*–oprL-gene forward: ACC CGA ACG CAG GCT ATG, reverse: CAG GTC GGA GCT GTC GTA CTC), 0.1 μM of hydrolysis probe 6FAM-5’ AGAAGGTGGTGATCGCACGCAGA3’-BlackBerry Quencher) and 2.5 μl of DNA-extract, for a final volume of 10 μl. PCR annealing temperature was 55°C. A standard tenfold dilution series was prepared using a quantified DNA of *P*. *aeruginosa* strain PA14 for which the genome number was estimated and translated into CFU. This standard dilution series was used in qPCR analysis to construct a standard curve, which enabled to calculate the number of cells (in CFU) in the samples on the basis of the obtained Ct-values.

The qPCR targeting *A*. *fumigatus* were performed for both PMA-treated and untreated samples by real-time PCR performed with a 5-μl DNA volume on a LightCycler instrument (Roche, Meylan, France), as previously described [18].

### Metagenomic library preparation, sequencing and phylogenetic assignation

The hypervariable V3–V5 regions of 16S-rRNA gene were amplified using the primers: For16S_519, CAGCMGCCGCGGTAATAC and the reverse primer Rev16S_926, CCGTCAATTCMTTTGAGTTT. The ITS2 loci were amplified using the forward primer GATGAAGAACGYAGYRAA, and reverse primer RBTTTCTTTTCCTCCGCT [19].

Indexed amplicon libraries were clonally amplified with Ion PGM^™^ Template OT2 400 Kit and the Ion OneTouch^™^ ES Instrument (Ion Torrent), and sequenced through PGM, Ion Torrent (Life Technologies).

Raw data analysis was performed using a home-made pipeline composed of open-source softwares (Mothur [20], Esprit-tree [21], biome format [22]), databanks and home-made Perl/python scripts, which were all implemented in Galaxy [23]. Briefly, the first step corresponded to a preprocessing step producing a curated and filtered collection of reads using Mothur tools [20]. All reads shorter than 150 bases were removed. The remaining sequences were trimmed to remove the erroneous homopolymers generated by the Ion Torrent PGM sequencer, with a maximum limit for homopolymer length set to 20. Once this filtering was applied, duplicated sequences were grouped to save computing time during the alignment and clustering steps. The phylogenetic analysis was based on 16S rRNA gene classification from RDP, Silva and GreenGene databases [24] and ITS2 gene classification from DBScreen database [25]. Sequences with alignments less than 100 bases were filtered out.

### Statistical analyses

Data were considered as paired PMA-treated and untreated samples to study PMA impact in the ecological characterization of a given sample. According to published data [26–28], fungal and bacterial communities of each sample were divided into sub-populations as follows: Abundant taxa were defined as genera with a relative OTU abundance greater than or equal to 1% and intermediate taxa as having a relative abundance of less than 1%.

Alpha diversity indexes (richness, Shannon-Wiener and Simpson’s indexes) were compared using Wilcoxon test. Beta diversity and OTU analysis were accessed using ANOSIM (analysis of similarity) method, PCA (principal component analysis), and the Bray-Curtis similarity index. To estimate similarity and changes in community composition of paired samples, we applied a hierarchical cluster analysis based on the relative abundance of taxa in the samples using Bray-Curtis similarity and a dendrogram inferred with the unweighted pair-group average algorithms. The clustering robustness was accessed by bootstrapping (10,000 replicates). The p values were corrected using the Bonferroni correction for multiple comparisons. Numerical variables were described as means and standard deviations or 95% confidence interval. p-values of less than 0.05 were considered as significant. All statistical analyses were performed using PAST software version 3.05 [29].

## Results

### Overall effect of PMA on the polymicrobial community in CF sputum samples

**Fig 1:**
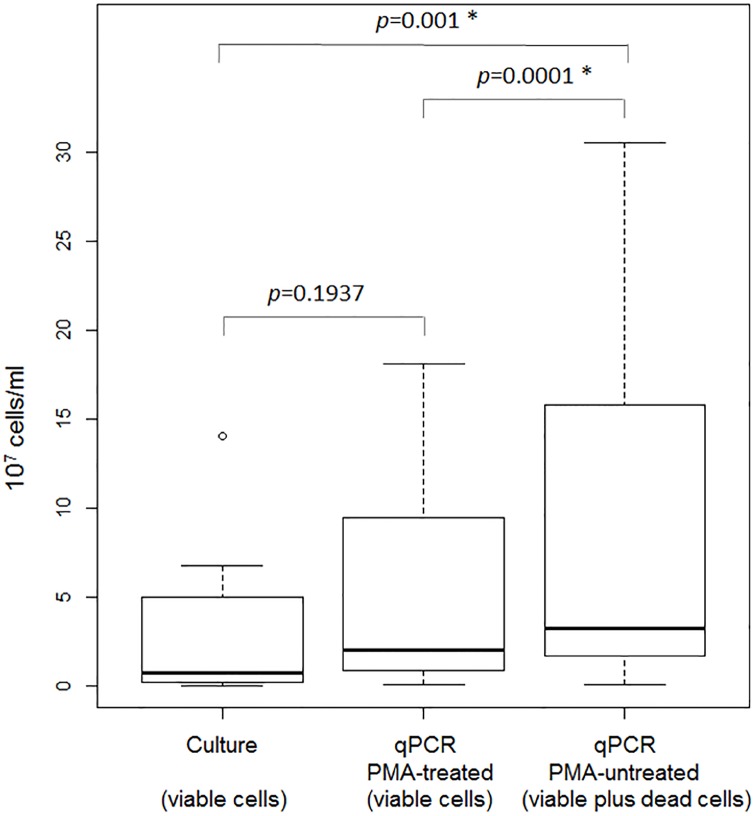
Comparison of the enumeration of *P*. *aeruginosa* cells between cultures and qPCRs of PMA-treated and untreated sputum samples.

The mean abundance of *P*. *aeruginosa* cells in PMA-untreated samples using qPCR (total DNA, 7.44 ± 9.21 x 10^7^ cells/ml) was significantly higher than that of PMA-treated samples (5.17 ± 6.50 x 10^7^ viable bacteria/ml; *p* = 0,00006 using pairwise t-test) and that of cultures (2.91 ± 3.98 x 10^7^ cells/ml), whereas *P*. *aeruginosa* abundances estimated by qPCR in PMA-treated and culture samples were not statistically different (Fig 1). For each pair of samples, qPCR results showed a regular positive difference between the two conditions, in agreement with the expected PMA effect.

Regarding HTS raw data (ENA registered http://www.ebi.ac.uk/ena/data/view/PRJEB14967, and S1 Table), the average total number of reads (both fungal plus bacterial reads) of PMA-treated samples was slightly higher than that of untreated ones (respectively 176,135 ± 136,953 and 168,434 ± 178,433 reads). The mean length of sequences from PMA-treated samples was shorter than that of untreated samples (respectively 323 ± 22 and 328 ± 16 bp) without any statistical significance.

**Fig 2:**
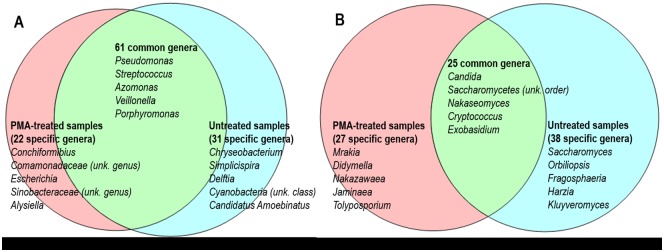
Venn diagram representing number of shared and specific bacterial (A) and fungal (B) genera between PMA-treated and untreated samples. In each case, the first 5 most prevalent genera are listed.

**Fig 3:**
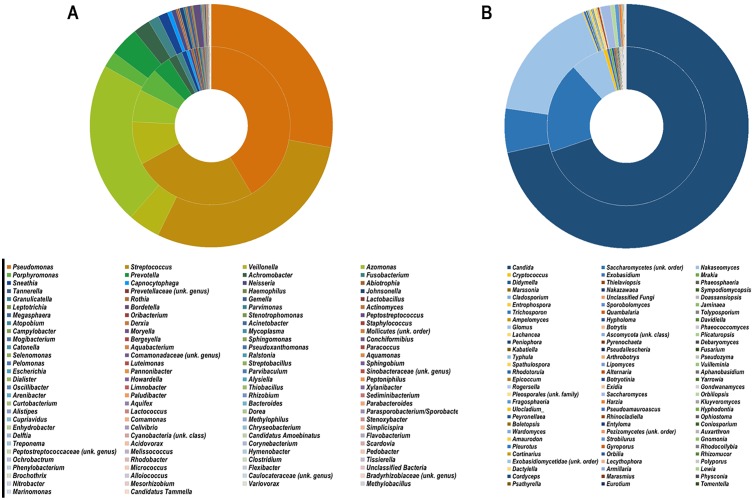
Global distribution of bacterial (A) and fungal (B) genera between PMA-treated (inner ring) and untreated groups (outer ring). All genera are ordered in descending relative abundance.

For each sputum sample, we characterized the composition and diversity of fungal and bacterial microbiota. One and 5 pairs of samples were excluded from bacteriome and mycobiome analysis respectively since corresponding rarefaction curves and/or numbers of reads were inadequate to allow biodiversity comparison. Therefore, among the 15 paired samples, only 14were analyzed at the bacterial NGS level and 10 at the mycological NGS level. The 14 paired samples (n = 28) exhibited a total of 763,802 bacterial reads corresponding to 114 bacterial genera, and the 10 paired samples (n = 20) exhibited 655,284 fungal reads corresponding to 90 fungal genera (Figs 2 and 3). As the patient status regarding *Aspergillus* colonization was not expressly known, we retrospectively assessed for *A*. *fumigatus* occurrence using qPCR in order to validate the results of ITS2 deep sequencing-based method [18]. Only sputum samples from patient “G172” exhibited positive PCR results at the limit of detection threshold (40.1 ± 2.1 Ct) [18,30].

The number of bacterial sequences in the samples ranged from 1,560 to 55,495 reads with an average of 27279 ± 12673 (n = 28). The dominant genera co-presenting with *Pseudomonas* (34.9%) were *Streptococcus* (27.4%), *Azomonas* (13.8%), *Veillonella* (6.6%), *Prevotella* (3.8%), *Porphyromonas* (3.5%), *Achromobacter* (2.0%), *Fusobacterium* (1.3%) and *Sneathia* (1.1%) (Fig 3A). The mean number of fungal sequences was 32764 ± 39977 (n = 20). Dominant fungal genera were *Candida* (70.5%), genus belonging to *Saccharomycetes* (13.0%), and *Nakaseomyces* (11.3%) (Fig 3B).

The overall relative abundance of *Pseudomonas* genus increased from 27.8% in untreated samples to 41.4% in PMA-treated ones (Fig 3A), while the species *P*. *aeruginosa* was decreased after PMA-treatment, which may be linked to the antibiotic regimen (designed to kill *P*. *aeruginosa* cells but not all the cells of genus *Pseudomonas*). *Veillonella* also increased from 4.3% to 8.8%, and *Porphyromonas* from 2.1% to 4.8%. By contrast, a number of genera decreased in PMA-pretreated samples: in particular, the relative abundance of *Streptococcus* decreased from 29.3% to 25.5%, and that of *Azomonas* from 21.6% to 6.6%. The majority of these bacteria are described as susceptible to piperacillin/tazobactam. Regarding mycobiota overall data, the relative abundance of *Candida* and *Nakaseomyces* decreased from 71.5% to 69.7% and from 16.7% to 6.9% respectively (Fig 3B).

### Impact of PMA on biodiversity and similarity among fungal and bacterial communities

**Fig 4:**
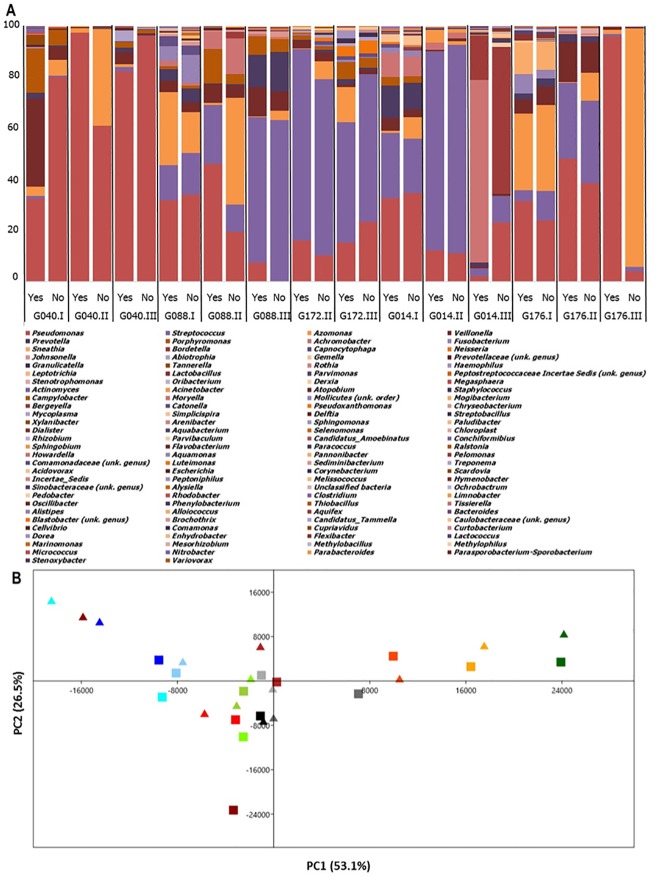
Effect of PMA on the composition of bacteriome of each sample. **(A)**: Relative abundance of bacterial genera of each sample (“yes” or “no” indicates the samples with or without PMA-pretreatment). **(B)**: PCA plot of the first two components of bacteriome of samples with and without PMA-pretreatment. Each marker represents treatment conditions (filled triangle symbols PMA-treated samples, filled square symbols untreated samples). Each color represents a given sputum sample (G040.I: light blue; G040.II: aqua; G040.III: dark blue; G088.I: yellow green; G088.II: chartreuse; G088.III: dark green; G172.II: orange; G172.III: dark orange; G014.I: grey; G014.II: dark grey; G014.III: black; G176.I: red; G176.II: firebrick; G176.III: dark red).

**Fig 5:**
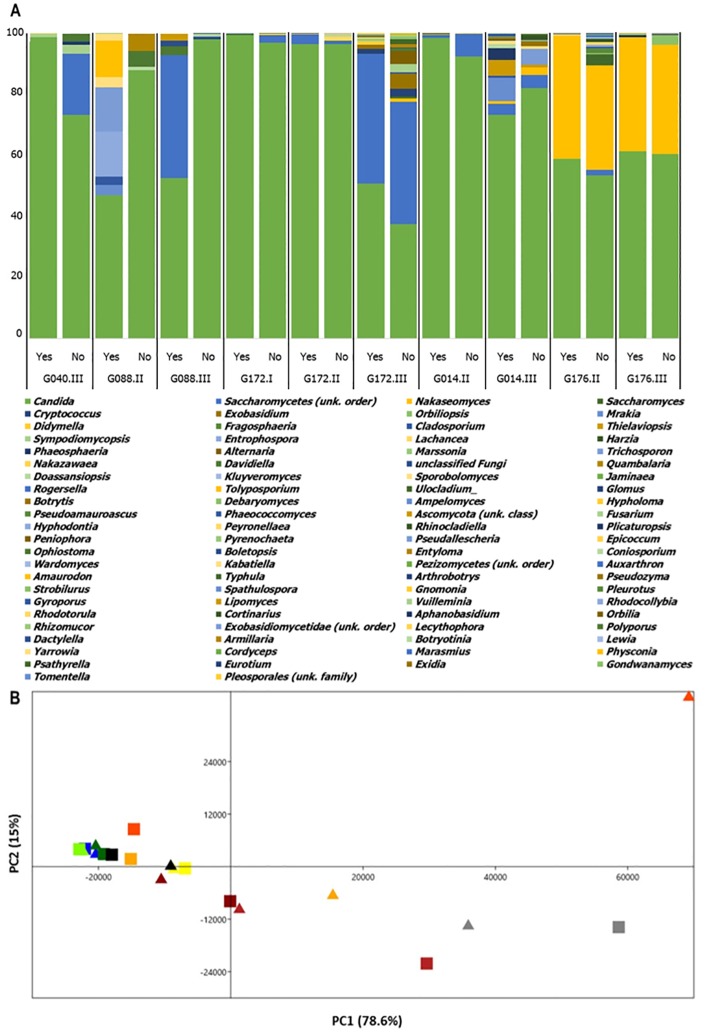
Effect of PMA on the composition of mycobiome of each sample. **(A)**: Relative abundance of fungal genera of each sample (“yes” or “no” indicates the samples with or without PMA-pretreatment). **(B)**: PCA plot of the first two components of mycobiome of samples with and without PMA-pretreatment. Each marker represents treatment conditions (filled triangle symbols PMA-treated samples, filled square symbols untreated samples). Each color represents a given sputum sample (G040.III: dark blue; G088.II: chartreuse; G088.III: dark green; G172.I: yellow; G172.II: orange; G172.III: dark orange; G014.II: dark grey; G014.III: black; G176.II: firebrick; G176.III: dark red).

PMA-treated and untreated groups shared 61 bacterial and 25 fungal genera (mostly referred as dominant genera). While a majority of known bacterial and fungal pathogens were shared by both types of samples (Fig 2), the bacterial or fungal genera referred as intermediate populations mainly belonged to the unshared populations (Figs 4A and 5A). For each sample pair, HTS results were highly congruent between the two conditions (with and without PMA-pretreatment) excepted for samples G014-III, G176-III, and G088-II (Figs 4 and 5). The genus numbers of both bacterial and fungal unshared populations were slightly higher in untreated samples than in PMA-treated ones, as confirmed by means of richness and biodiversity indexes (Table 1). More interestingly, we identified a significant difference in the intermediate bacterial population based on Simpson index (p = 0.03) and a trend toward significance based on Shannon index (p = 0.06) (Table 1). No significant differences in alpha diversity indexes of mycobiota data were observed (Table 1).

**Table 1:** Abundance and α-diversity comparison of bacteriome and mycobiome between PMA-pretreated and untreated samples.

Paired sputum samples	All populations			Abundant population (≥ 1%)			Intermediate population (<1%)		
	*PMA-treated	Un-treated	^μ^*p*	PMA-treated	Un-treated	*p*	PMA-treated	Un-treated	*p*
***Bacteriome analysis (Number of paired samples = 14)*:**									
**Total number of reads	28280 ±14356	26278 ±11194	*0*.*8*	27617 ±13955	25591 ±10873	*0*.*8*	663 ±538	686 ±490	*0*.*9*
**Richness**	20 ±8	22 ±11	*0*.*3*	6 ±3	6 ±3	*1*	13 ±7	16 ±9	*0*.*3*
**Shannon index**	1.2 ±0.6	1.2 ±0.6	*0*.*6*	1.1 ±0.6	1.1 ±0.6	*0*.*7*	1.8 ±0.6	2.0 ±0.6	*0*.*06*
**Simpson index**	0.5 ±0.3	0.5 ±0.2	*0*.*9*	0.5 ±0.3	0.5 ±0.3	*0*.*7*	0.8 ±0.2	0.8 ±0.1	***0*.*03***
***Mycobiome analysis (Number of paired samples = 10)*:**									
**Total number of reads**	36618 ±44508	28910 ±36879	*0*.*9*	36286 ±43812	28637 ±36386	*0*.*9*	332 ±763	273 ±741	*0*.*5*
**Richness**	9 ±6	12 ±7	*0*.*9*	6 ±4	8 ±4	*0*.*2*	3 ±3	4 ±5	*0*.*7*
**Shannon index**	0.7 ±0.5	0.7 ±0.5	*0*.*9*	0.6 ±0.5	0.6 ±0.5	*0*.*8*	0.6 ±0.6	0.4 ±0.3	*0*.*8*
**Simpson index**	0.3 ±0.3	0.3 ±0.2	*0*.*9*	0.3 ±0.3	0.3 ±0.2	0.9	0.3 ±0.3	0.4 ±0.3	*0*.*7*

*PMA, propidium monoazide;

^μ^***p***: p value (significant p value is bolded);

**Total number of reads and all diversity index expressed as mean and standard deviation.

Based on Bray-Curtis similarity calculation, the clustering of the entire communities (S1 Fig) was similar to the grouping of the abundant populations (S2 Fig) for both bacterial and fungal microbiotas. Only 2 pairs of samples still exhibited such a similarity for their bacteriome when the intermediate population was analyzed (S3 Fig).

PCA and outcome of the ANOSIM of the OTU data showed similar results: the paired samples (squares versus triangle in Figs 4B and 5B) still clustered relatively close to each other. The main bacteria explaining the variance for the first axis in the PCA plots were from OTUs of *Prevotella*, and of *Pseudomonas*, *Streptococcus*, *Veillonella* for the second axis (Fig 4B). The principal fungi explaining the variance for the first axis were from OTUs of genera belonging to *Candida*, *Saccharomycetes*, and *Nakaseomyces*. The main loadings of the second axis were determined by those of *Saccharomycetes* (unknown order and genus) (Fig 5B). ANOSIM method confirmed these findings, showing no significant differences between the PMA-treated sample data and the untreated ones (Table 2). These beta-diversity results are consistent with those of the alpha-diversity indexes.

**Table 2:** *p-values* and *r-values* of similarity analysis (one-way ANOSIM) of the bacterial and fungal OTU data.

OTU data	Population and sub-populations	^a^*p*-values	^b^*r*-values
**Bacteriome**	Global population	0.52	-0.011
	Abundant population	0.52	-0.010
	Intermediate population	0.97	-0.066
**Mycobiome**	Global population	0.84	-0.062
	Abundant population	0.84	-0.062
	Intermediate population	0.56	-0.009

^a^ *p- values* indicate the significant differences between PMA-treated and untreated groups;

^b^ *r-values* indicate dissimilarity between groups as follow: the higher the *r-value* is, the more dissimilar the compared groups are.

## Discussion

Chronic pulmonary colonization with recurrent infective exacerbations, caused by intercurrent bacterial, viral and/or fungal infections, produces an irreversible decline in lung function and early death in CF patients [1,7]. Although a diversity of bacterial species can be isolated from the CF airways, *P*. *aeruginosa* is the most common CF pathogen able to develop chronic infections with acute exacerbations [1,31,32]. Once established, chronic *P*. *aeruginosa* infections are difficult to treat with antibiotics and the pathogen is virtually never eradicated due to biofilm formation [31]. Furthermore, *P*. *aeruginosa* biofilm may interact with fungi, displaying an increase in mutability in mixed biofilms [33,34]. *P*. *aeruginosa* consortium is also modified according to a Climax/Attack model when exacerbation occurred [3,4]. In this model, fermentative anaerobes are hypothesized to be the core members of the Attack Community and responsible for exacerbation [4]. In this context, analyzing microbial community of CF respiratory tract by using HTS coupled with PMA-pretreatment may facilitate the detection of both viable bacteria (including obligate anaerobes) and fungi potentially involved in the exacerbation, without underestimating or overestimating DNA quantifications and microbial community compositions.

While PMA-pretreatment has been combined with various molecular techniques and successfully applied for discriminating between living and dead cells in microbiology [6,8,10–17,35], it has been combined to pyrosequencing in only few studies to analyze the bacterial community of environmental and human samples [6,11–15]. Given the importance of detecting the whole viable members at metacommunity level, we studied both respiratory mycobiome and bacteriome of CF patients colonized with *P*. *aeruginosa* during exacerbation using PMA-pretreatment combined with HTS. Since the mycobiome in general and the lung mycobiome in particular have not been fully investigated [36], this study provides the first opportunity to focus on the PMA-pretreatment effect on this understudied eukaryotic community and its co-presence with the bacteriome in human airways during acute pulmonary exacerbation.

Apart from *Pseudomonas* identified as dominant bacterial genus (in agreement with our selection criterion of patients chronically colonized with *P*. *aeruginosa*), our results (Fig 3A) confirmed the core taxa of bacteria in CF respiratory microbiome previously described (for review: [5]). The 9 most abundant genera contributed 94.4% of the total number of sequences while the rest of more than a hundred genera contributed only a small proportion of the total OTU number (Fig 4A). Several bacterial species detected in the CF respiratory microbiome here were also considered as the most frequent oral commensal bacteria such as *Streptococcus*, *Prevotella*, *Porphyromonas*, *Rothia*, *Tannerella*, *Fusobacterium*,… This result confirmed the close relation between respiratory and buccal microbiotas due to microaspirations of salivary and potentially explained by the neutral dispersion model recently proposed [37–39]. In addition regarding the co-presence of *Pseudomonas* and oral commensal bacteria, there is recent evidences showing that the oral commensal streptococci could modulate the growth of *Pseudomonas* in CF disease condition [40].

Similar to bacteriome results, 3 fungal genera accounted for 94.7% of the total number of fungal OTUs. Among them, *Candida* genus (including *Candida albicans* (52.3%)) was the dominant genus of the CF respiratory mycobiome in our study (Figs 3B and 5A). *C*. *albicans* has been frequently isolated with high prevalence in CF sputa, even if its role in pathogenesis and clinical CF evolution is still matter of debate (for review: [36,41]). There is also evidence showing the co-presence of *C*. *albicans* and *P*. *aeruginosa* in patients’ respiratory tract as an opportunistic damaging association [33]. The remaining fungal genera represented a limited number of OTUs, and confirmed the core taxa of lung mycobiome previously described [25,42–44]. Similar to bacteriome analysis, the high abundance of *Candida* and *Saccharomycetes* observed here might also refer to the overlap with the buccal mycobiota in agreement with published data [37–39]. Interestingly, our study identified an important proportion of *Nakaseomyces delphensis* (11.3%) in 7 of total 20 samples from 3 out of the 5 patients. This cultivable non-pathogenic yeast is closely related to *Candida glabrata* [45]. To our knowledge, it has never been detected in human respiratory tract. These results underline the large exposure of lungs to environmental microorganisms that may play a role in chronic respiratory diseases such as CF, and highlight the importance of analyzing the whole respiratory microbiota, which could be a reservoir of diverse microorganisms yet to be identified or yet to be classified as pathogens in CF (such as *C*. *albicans*).

As expected, PMA-pretreatment reduced significantly the amplifiable amount of *P*. *aeruginosa* DNA and the number of viable cells (Fig 1), in agreement with patient treatment and published data [6,17]. This result is also compatible with the ability of *P*. *aeruginosa* to form biofilms that contain a higher proportion of dead cells, and are observed in lungs of CF patients chronically colonized with *P*. *aeruginosa* as our patient population [6,17,35]. As OTU numbers were slightly higher (Table 1) and the mean length of sequencing reads was shorter in PMA-treated samples than in untreated ones, we could refer to some limitations of the PMA-treatment technique recently described [13,46,47]. Various factors affecting the influence of PMA treatment on the results of both qPCR and HTS (type of samples, PMA concentration, PMA incubation time, light source and exposure time, length of target gene, pH) have been proposed [13,46,47]. We principally followed the steps that were in agreement with an optimal effect of PMA-pretreatment [16,17]. The estimated numbers of viable cells in our samples were more than 10^7^ cells/ml, in agreement with the cutoff of 10^5^ cells/ml recently proposed to get the most adequate effect of PMA-treatment [35]. Furthermore, the mean length (>300 bp) of sequencing reads from both PMA-treated and untreated samples was long enough to show an accurate detection of viable cells [13]. The use of multiple viability filters (sample treatment with DNase/Proteinase K or metabolic and enzyme activity estimations) has also been suggested [11].

As the present study has some limitations such as a modest sample size (15 samples from CF patients with acute pulmonary exacerbation, without a control group composed of CF patients clinically stable) or the absence of mycology culture records that have limited the data analysis, further studies are now warranted to fully evaluate the efficiency of PMA coupled with HTS.

Besides, the "rare” or “intermediate” population has recently become an emerging concept in ecology, which has been particularly studied in sea-water samples [27,28,48,49]. Despite different cutoffs used to differentiate the minority taxa (“rare” to “intermediate” taxa), all these studies have shown that minority populations vary more than majority ones and that minority taxa play a crucial role in the ecosystem stability [27,28,48,49]. We observed a significant difference in the diversity of intermediate bacterial populations between the two groups of samples (Table 1). This result is in agreement with Rogers et al.’s study which also evaluated the effect of PMA-treatment on *P*. *aeruginosa* abundance through qPCR and on the diversity of the lung bacteriome from CF patients who were judged to be clinically stable at the sampling time [6]. While Rogers et al. found that PMA-treatment resulted in an increase in community evenness driven by an increase in diversity of rare community members, we identified a decrease in community diversity of intermediate population. Similar to this study, and despite the two different types of CF populations, our results suggest that there was no significant difference in the entire bacterial community but PMA-pretreatment could significantly influence the apparent composition of “satellite” taxon groups analyzed by HTS. Several other studies using HTS compared the bacterial profiles of PMA-treated and untreated environmental samples and showed significant changes in the bacteriome structure [11–14]. Whereas Vaishampayan and colleagues [12] demonstrated a difference in the bacteriome structure of environmental samples with and without PMA-pretreatment using PCA, we found both PMA-treated and untreated samples situated virtually next to each other (Fig 4B), in agreement with Exterkate and colleagues’ results, studying oral cavity samples [14].

Regarding the mycobiome, we could not establish any significant difference of abundance or biodiversity between PMA-treated and untreated samples (Table 1). Despite some changes in the mycobiota compositions (Fig 5A and S1 Fig), no significant difference in both abundant and intermediate populations was observed. To the best of our knowledge, this is the first study focusing on the effect of PMA with regard to airway mycobiome characterization by HTS, which limits the comparison and discussion of our findings. However, PMA-treatment has been recently coupled with HTS to investigate the fungal burden of room dust samples [15]. The authors found remarkable differences between the PMA-treated and untreated samples, and indicated the presence of a large proportion of pathogenic fungal genera such as *Aspergillus*, including *A*. *fumigatus* which represents the most frequent pathogenic mold isolated in CF respiratory samples [41]. We identified *Candida*, *Malassezia*, *Alternaria*, and *Fusarium*, but OTUs belonging to *Aspergillus* genus were not isolated. This result could be explained by some bias in DNA extraction or amplification. As we were able to amplify DNA from other phylogenetically close filamentous genera such as *Fusarium*, DNA extraction method should be sufficient to breakdown fungal cell walls. The primers we used were shown to be highly efficient for amplification of diverse fungal species including those of the Ascomycota, Basidiomycota and non-Dikarya [19]. This suggests that *A*. *fumigatus* was indeed not present in our samples, as confirmed with the *Aspergillus* qPCR results.

Considering the co-evolution and co–exclusion of core taxa, our results suggest that PMA-pretreatment explains better the physiopathology of these core taxa and their interactions in the CF lung environment. We report a proportion of *Pseudomonas* increasing from 27.8% to 41.4% of total reads in the PMA-treated group, in agreement with Rogers et al.’s results [6]. In parallel, OTUs of *Streptococcus*, *Azomonas*, and *Candida* genera decreased (Fig 3). These microbial proportions modified between the PMA-treated and untreated groups may reflect the behaviors of microbial communities within CF lungs: *(i)* Decrease in pH, low concentration in oxygen, high level of mucus, and bioavailability of metabolites such as alanine and lactate represent optimal conditions for *P*. *aeruginosa* growth, especially as biofilm consortium that PMA-treatment put prominently by differentiating viable to dead cells (for review: [39]. *(ii)* By providing an appropriate competitive niche for *Pseudomonas* genus, other genera such as *Streptococcus* and *Azomonas* may lose their survival advantages in CF lungs, a hypothesis in agreement with Filkins et al.’s analysis [50] which concluded that *Streptococcus* may play a central role in the stability of the lung microbiome. *(iii)* At the cross-kingdom communication level, lung interactions between *Candida* (especially *C*. *albicans*) and *Pseudomonas* or *Streptococcus* have been described [33,51,52]. There is evidence that *Candida* and *Pseudomonas* coexist in biofilms with both negative and positive association. Roux et al. [51] showed that rats inoculated with *P*. *aeruginosa* developed pneumonia only in the presence of viable *C*. *albicans*. *P*. *aeruginosa* secretes phenazines which are toxic to yeast and hyphal forms of *C*. *albicans*. It also produces a quorum-sensing which is similar to farnesol, a molecule secreted by *C*. *albicans* to regulate hyphal growth (for review: [53]). All these interactions may contribute to the formation of a specific microbiome in CF patients’ lungs able to face host-immune response and antimicrobial treatment. Our findings add support to the complex interaction especially during exacerbation between typical pathogens and microbiota, such as the association between *P*. *aeruginosa* and anaerobes, and/or fungi.

## Conclusion

The rationale of this study was whether PMA-pretreatment had an effect on the measured bacterial and fungal compositions, presumably resulting in a more accurate account of the viable microorganisms in CF lung microbiota. While culture-independent methods such as HTS are not able to discriminate dead from viable cells, culture dependent methods allow us to identify viable microorganisms, but not non-cultivable ones. PMA combined with HTS is therefore believed to be a worthy perspective solution that allowed us to show that during acute exacerbation the CF airway microbiome is diverse and largely made up of viable community members. According to our results and published ones [6], PMA-treatment highlights changes in the relative abundance of OTUs in the CF sputum samples which can help for respiratory microbiota analysis without overestimating the abundance of each viable microorganism. Due to its time consumption and cost, this technique should be limited to cases that are difficult to manage. Improvement of the clinical status is the main objective when CF patients chronically colonized with *P*. *aeruginosa* receive antibiotic treatment to face acute exacerbation. Therefore, including PMA-pretreatment in the HTS sample analysis may be considered as a second line diagnostic tool used when the patient does not respond well to antimicrobial treatment, and/or when biofilm consortium is suspected; these two clinical situations could benefit from an accurate estimation of the “core” or abundant viable community members during exacerbation.

Further studies combining HTS analysis to viable microorganism differentiations are warranted to open new strategies for CF patient management. Given our results and the ability of filamentous fungi to cause acute pulmonary exacerbation, lung mycobiota analysis have to be included in these studies to give a realistic composition of the CF lung microbial community that may shape a local response and explain a specific clinical evolution.

## Supporting Information

S1 Fig Individual comparison of *P*. *aeruginosa* abundances estimated by qPCR in PMA-treated (circle in black) and untreated (circle in red) samples. (TIF)

S2 Fig Dendrograms representing the similarity between the composition of bacteriome and mycobiome from individual samples in all taxa. Clustering is based on Bray Curtis similarity distance matrix (bootstrap 10000 replicates). Bootstrap values (in percentages) are given at the nodes.
(TIF)

S3 Fig Dendrograms representing the similarity between the composition of bacteriome and mycobiome from individual samples in abundant taxa (≥1%). Clustering is based on Bray Curtis similarity distance matrix (bootstrap 10000 replicates). Bootstrap values (in percentages) are given at the nodes.
(TIF)

S4 Fig Dendrograms representing the similarity between the composition of bacteriome and mycobiome from individual samples in intermediate taxa (<1%). Clustering is based on Bray Curtis similarity distance matrix (bootstrap 10000 replicates). Bootstrap values (in percentages) are given at the nodes.
(TIF)

S1 Table Repartition of reads at the bacterial and fungal genus level for each sputum sample treated and un-treated with PMA. (XLSX)
