# Supplementary figures and images for: Effects of Propidium Monoazide (PMA) Treatment on Mycobiome and Bacteriome Analysis of Cystic Fibrosis Airways during Exacerbation
Source: PLoS One. 2016 Dec 28;11(12):e0168860. doi: 10.1371/journal.pone.0168860 (PMC5193350; doi:10.1371/journal.pone.0168860)

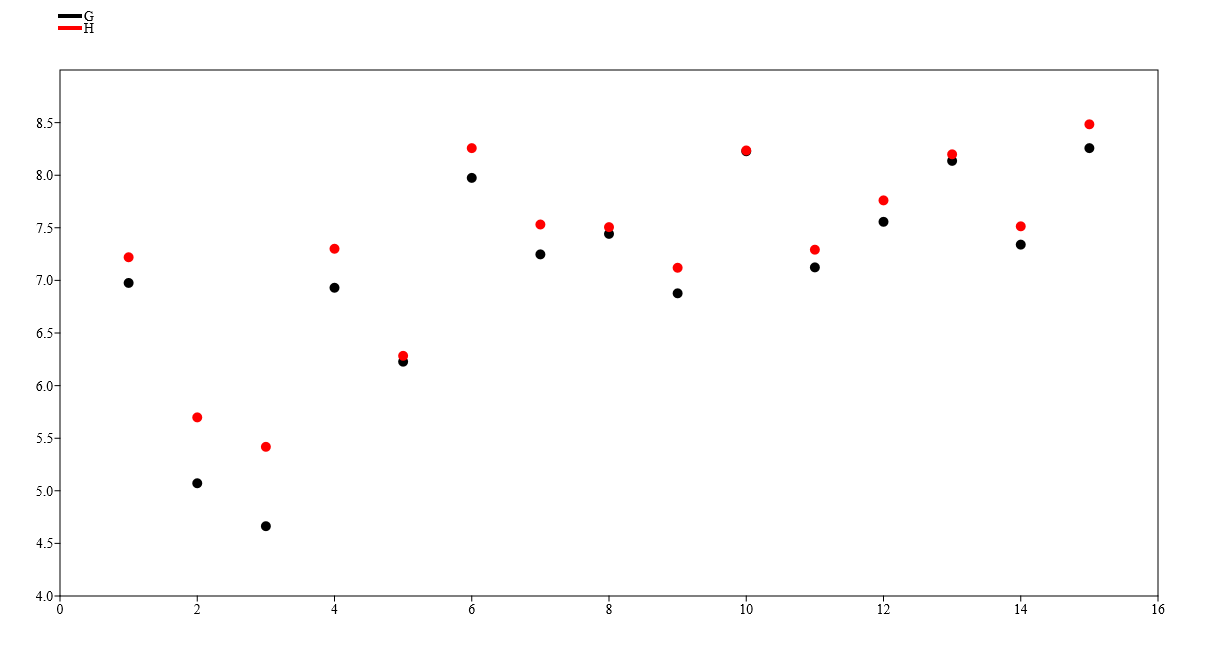

Supplement: S1 Fig — (TIF) [file pone.0168860.s001.tif]

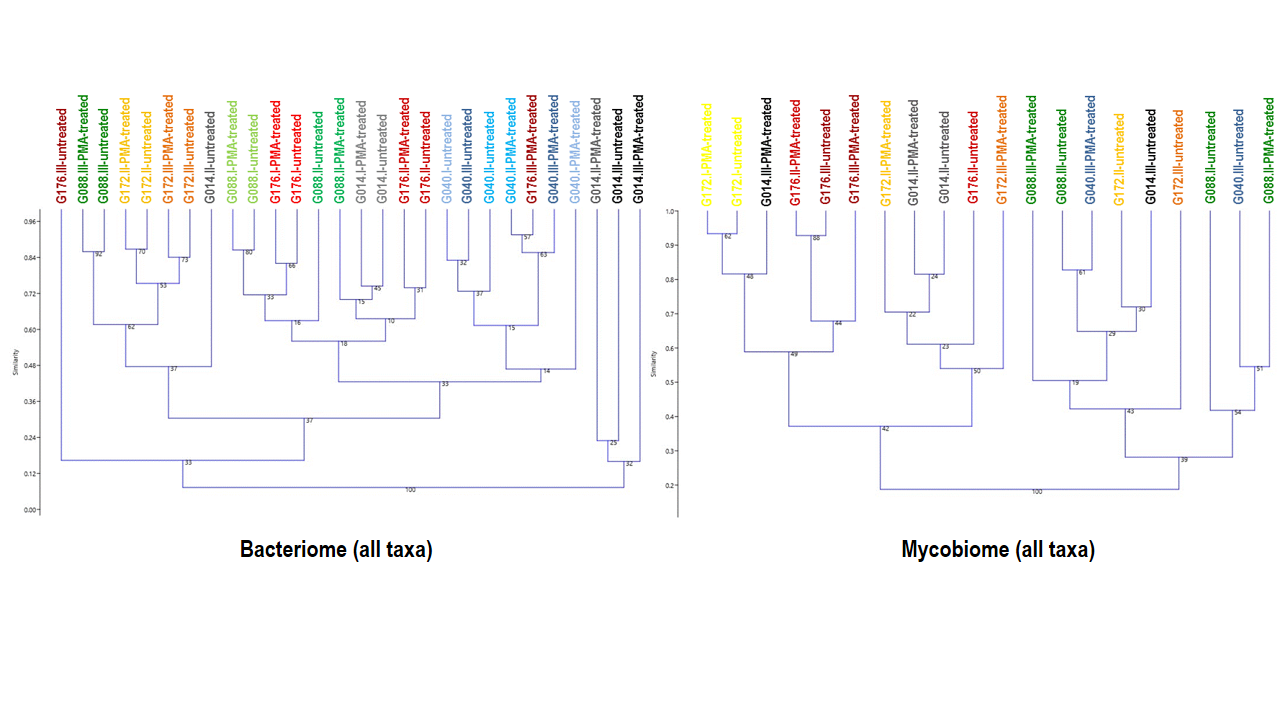

Supplement: S2 Fig — Clustering is based on Bray Curtis similarity distance matrix (bootstrap 10000 replicates). Bootstrap values (in percentages) are given at the nodes. (TIF) [file pone.0168860.s002.tif]

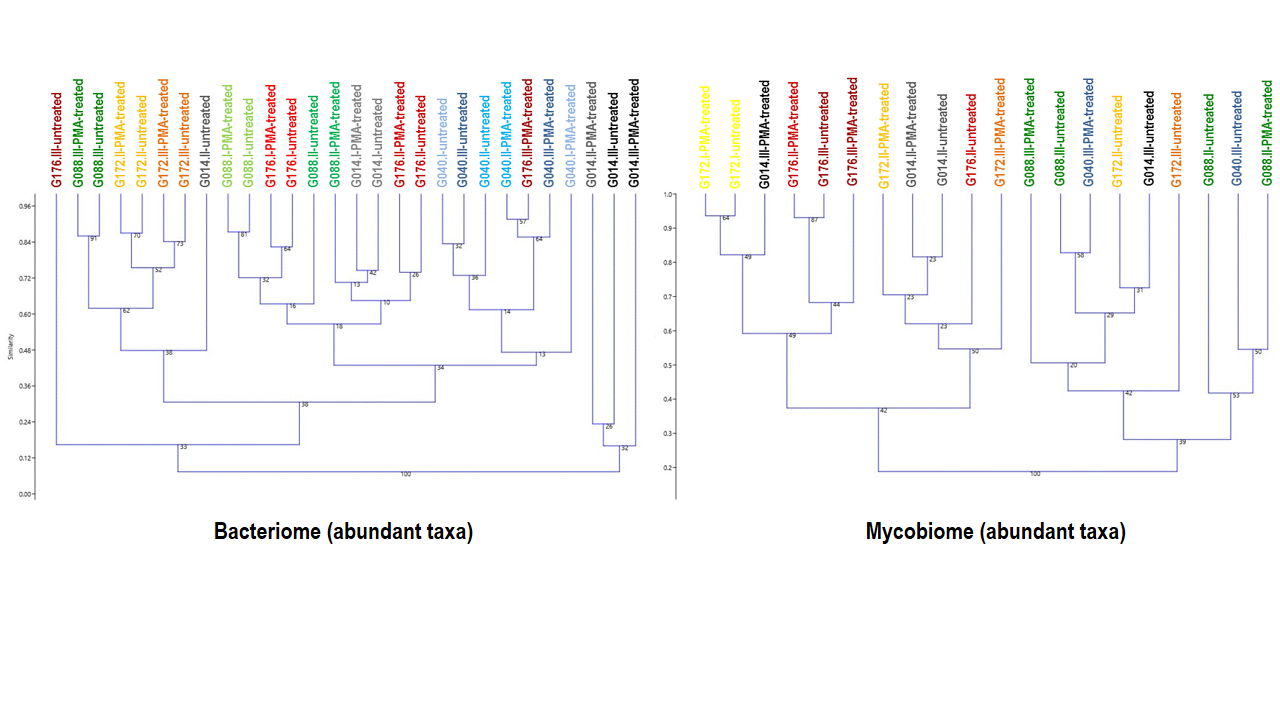

Supplement: S3 Fig — Clustering is based on Bray Curtis similarity distance matrix (bootstrap 10000 replicates). Bootstrap values (in percentages) are given at the nodes. (TIF) [file pone.0168860.s003.tif]

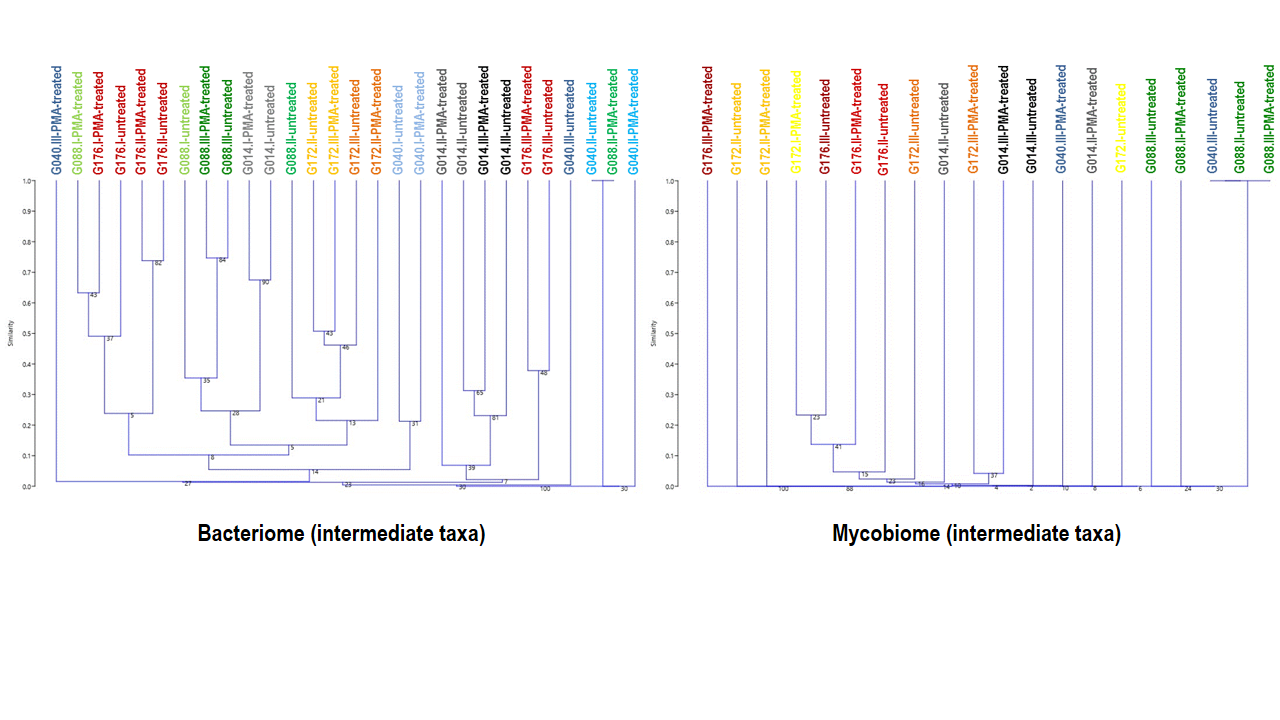

Supplement: S4 Fig — Clustering is based on Bray Curtis similarity distance matrix (bootstrap 10000 replicates). Bootstrap values (in percentages) are given at the nodes. (TIF) [file pone.0168860.s004.tif]
